# Supplementary material for: Microbial community pattern detection in human body habitats via ensemble clustering framework
Source: BMC Syst Biol. 2014 Dec 8;8(Suppl 4):S7. doi: 10.1186/1752-0509-8-S4-S7 (PMC4290728; doi:10.1186/1752-0509-8-S4-S7)
Supplement: Additional file 2 — Supplementary Material. The file presents several figures, tables and additional experimental results mentioned in this paper, including: (1) the efficiency of GPU-meta-storm algorithm; (2) evaluation of four base clustering results; (3) sensitivity study of phylogenetic structure similarity on microbial network. [file 1752-0509-8-S4-S7-S2.docx]

**Supplementary Material**

## Microbial community pattern detection in human body habitats via ensemble framework

Peng Yang, Xiaoquan Su, Le Ouyang, Hon-Nian Chua, Xiao-Li Li, Kang Ning^§^

^§^Corresponding author

Kang Ning: email: [ningkang@qibebt.ac.cn](mailto:ningkang@qibebt.ac.cn)

1. **Efficiency of GPU-meta-storm algorithm**

To study the total running time and speed up achieved by our method (GPU-Meta-Storms), we tried both the single core and multiple cores (with 16 threads) and for GPU we used the block size of (8*8). The similarity matrix of all datasets in our experiment is shown in **Table S1**. The performance in **Figure S1** shows that building the same similarity matrix by GPU computation has a maximum speed up of 3905 times compared to single core CPU and 593 times compared to 16-core CPU, which achieved the construction of similarity matrix for 1920 metagenomic samples within 4 minutes by GPU-Meta-Storms on Tesla M2075. Furthermore, the high speed-ups can not only largely eliminate the running time in computing the similarity of metagenomic data, but also enable in-depth data mining among massive microbial communities.

**Table S1: The sample features of each dataset from [1]**

| Dataset | # of samples | Total data size (Mb) |
| --- | --- | --- |
| Dataset 1 | 8 | 53 |
| Dataset 2 | 64 | 337 |
| Dataset 3 | 128 | 637 |
| Dataset 4 | 256 | 1331 |
| Dataset 5 | 512 | 2663 |
| Dataset 6 | 1024 | 5222 |
| Dataset 7 | 1920 | 9216 |

All experiments of this work are performed on a rack server with dual Intel Xeon E5-2650 CPU (16 cores in total, 2.0GHz), 64GBDDR3ECC RAM, NVIDIA M2075 GPU (448 stream processors and 6GBGDDR5 on board RAM) and 1TB hard drive in RAID 1.


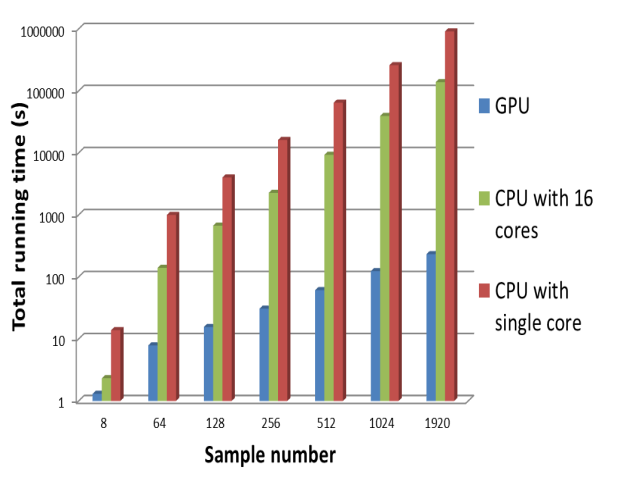
**
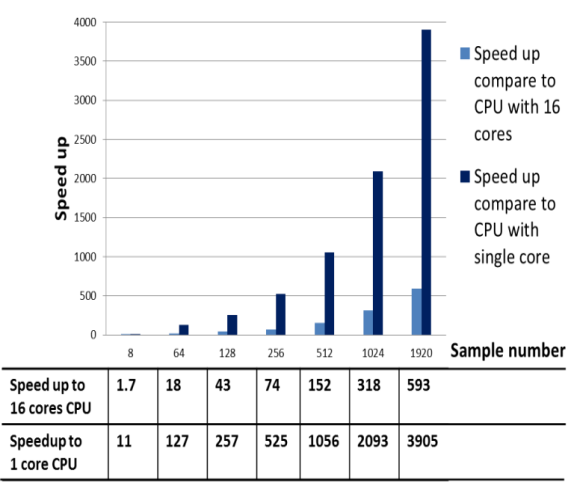
**

(A) (B)

**Figure S1: (A) Overall running time and (B) speed-up for similarity matrix computing by CPU and GPU.** From the results, we observed that building the same similarity matrix by GPU computation has a maximum speed up of 3905 times compared to single core CPU and 593 times compared to 16-core CPU, which achieved the construction of similarity matrix for 1920 metagenomic samples within 4 minutes by GPU-Meta-Storms [2] on Tesla M2075. Furthermore, the high speed-ups can not only largely eliminate the running time in computing the similarity of metagenomic data, but also enable in-depth data mining among massive microbial communities.

1. **Evaluation of four base clustering results**

**Enrichment analysis on predicted clusters**:

(A) Firstly, a global enrichment profile containing the enrichment results of all clusters are computed (**Table S2**). Results have shown that the most common features to be enriched are *f_Male_* and *f_Skin_*. And for those clusters enriched in both *f_Male_* and *f_Skin_*, the feature *f_Male&Skin_* is also enriched. This is especially true for clusters by k-mean and density-based method. Additionally, it was observed that for most of the clusters, the difference between the highest and second highest enrichment values are very large, indicating that most of these clusters are enriched by just one feature or two conditional independent features (such as male and saliva, as two different types of features).

(B) As shown in **Figure S2**, hierarchical clustering successes in predicting clusters enriched with gut and oral cavity, reflecting these clusters are very dense modules, and gut/oral cavity samples common species. On the contrary, four clustering models consistently predict one or two skin cluster in all experimental setting, inferring skin samples have many cluster patterns with diverse microbial structures. Additionally, we observed that for both k-means and density-based method, the enrichments of gender (male) and habitats (gut and saliva) could be observed, indicating that the results by these two methods were quite consistent.

Furthermore, contract to variable habitat enrichments on same batch clusters, the gender is nearly unchangeable on clusters. It is also observed that on overall cluster groups, combined taxonomy enrichment is always poorer than that of habitats.

## Table S2: The enrichment analysis of clusters generated from four clustering approaches

The number of initial random point K=6, thus 6 clusters per method. Enrichment values above 0.85 are annotated in black.

| Environmental factors | Hierarchical | | k-means | | Expect-Max | | density-based | |
| --- | --- | --- | --- | --- | --- | --- | --- | --- |
|  | K=6 | K=9 | K=6 | K=9 | K=6 | K=9 | K=6 | K=9 |
| ***f_Male_*** | 0.690 | 0.768 | **0.958** | 0.721 | 0.807 | 0.627 | 0.691 | **0.899** |
| ***f_Female_*** | 0.310 | 0.232 | 0.042 | 0.279 | 0.193 | 0.373 | 0.309 | 0.101 |
| ***f_Gut_*** | 0.001 | 0.003 | 0.021 | **0.989** | 0.136 | 0.001 | 0.361 | 0.066 |
| ***f_Skin_*** | **0.989** | 0.132 | **0.965** | 0.087 | 0.747 | **0.990** | 0.630 | **0.884** |
| ***f_Oral Cavity_*** | 0.010 | **0.865** | 0.014 | 0.022 | 0.117 | 0.009 | 0.008 | 0.050 |
| ***f_Male&Gut_*** | 0.001 | 0.004 | 0.007 | 0.712 | 0.014 | 0.001 | 0.264 | 0.015 |
| ***f_Male&Skin_*** | 0.687 | 0.121 | 0.937 | 0.007 | 0.719 | 0.624 | 0.425 | **0.849** |
| ***f_Male&Oral Cavity_*** | 0.002 | 0.643 | 0.014 | 0.002 | 0.074 | 0.001 | 0.002 | 0.035 |
| ***f_Female&Gut_*** | 0.000 | 0.100 | 0.014 | 0.277 | 0.122 | 0.100 | 0.097 | 0.051 |
| ***f_F&Skin_*** | 0.302 | 0.011 | 0.028 | 0.002 | 0.027 | 0.366 | 0.205 | 0.040 |
| ***f_Female&Oral Cavity_*** | 0.007 | 0.221 | 0.000 | 0.000 | 0.044 | 0.007 | 0.006 | 0.010 |


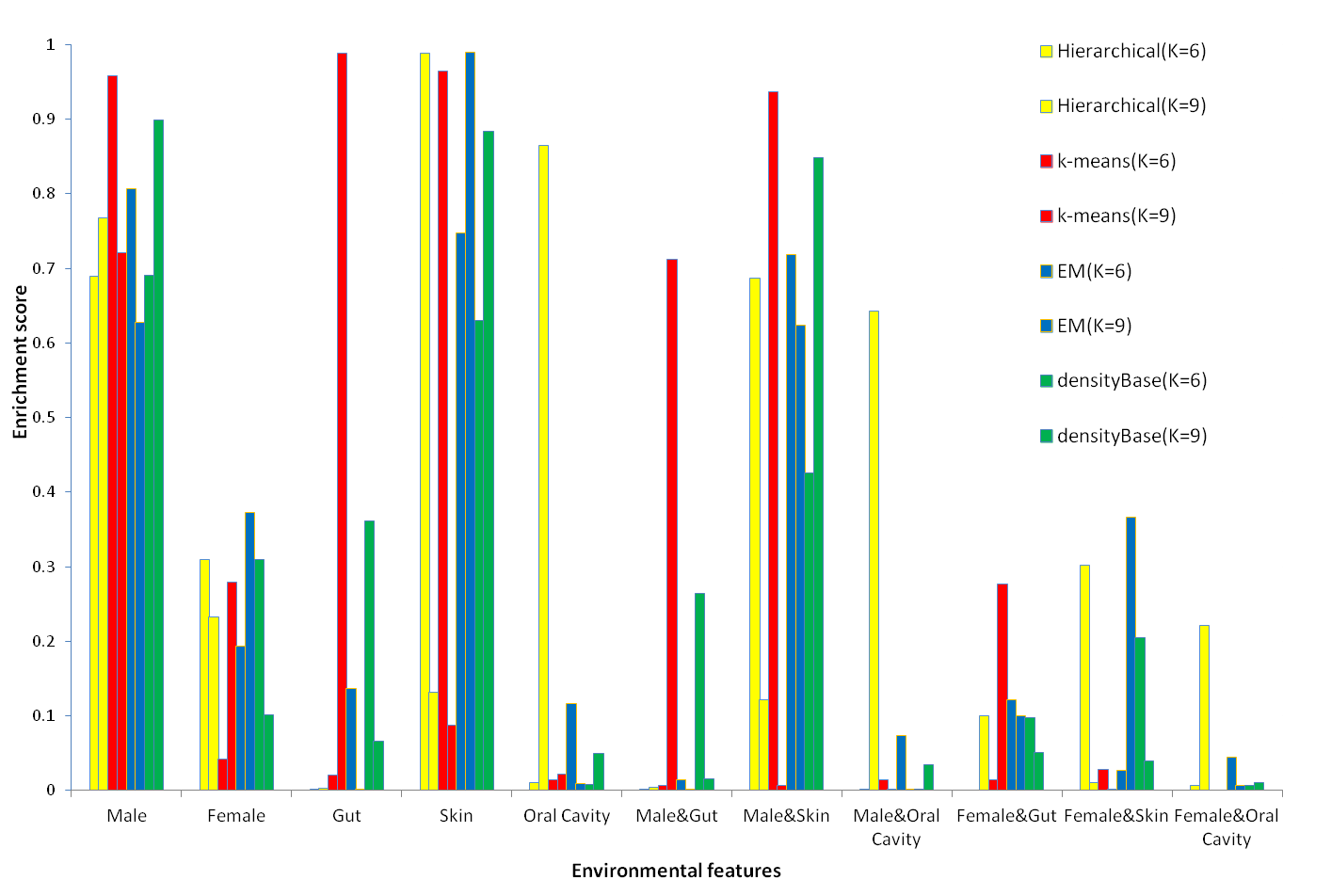


**Figure S2:** **Enrichment analysis on the same groups of clusters.** K is defined as number of clusters generated by base approaches. Habitat enrichment value equals to percentage of one habitat samples in one cluster.

**Enrichment analysis of “common” clusters**: Enrichment analysis is also proposed on “common” clusters by different clustering methods. Jaccard coefficient is applied to evaluate the similarity/overlapping of two clusters. Note that we only process large-sized clusters with at least 100 samples. **Table S3** shows that three “common” cluster sets are detected by all 4 clustering methods in our experiment. Each cluster is annotated by its clustering approach and parameter setting. **Table S3** also presents cluster size and cluster density of each common clusters in column 2 and 3. Then we apply enrichment analysis onto these three cluster sets and list the significant features (enriched scores > 0.9) in column 4 of **Table S3**. We observe that the size of skin clusters is much larger than that of other two taxonomy clusters. In addition, Gut clusters are always denser than skin and oral cavity clusters. Moreover, *Habitat* features play an important role on forming the clusters on the similarity network. Although four clustering approaches build the clusters with various measurements, these common cluster groups are all enriched with one of the habitats (skin, oral cavity and gut), indicating that in the similarity network, samples with identical habitats tend to interact with each other to form the modules to perform biological functions, while gender of the host seems to have little effect on this common cluster pattern.

## Table S3: Common clusters among four clustering models

| Common clusters (JC> 0.8) | Cluster Size | Cluster Density | Enriched features (score>0.9) |
| --- | --- | --- | --- |
| Hierarchical(K=6) | 722 | 0.910 | Skin |
| K-mean(K=9) | 584 | 0.930 |  |
| Densitybased(K=9) | 568 | 0.931 |  |
| EM(K=6) | 422 | 0.937 | oral cavity |
| Hierarchical(K=6) | 511 | 0.916 |  |
| K-mean(K=9) | 424 | 0.932 |  |
| Density based(K=9) | 401 | 0.938 |  |
| EM(K=6) | 357 | 0.939 | gut  male&gut |
| Hierarchical(K=9) | 382 | 0.907 |  |
| K-mean(K=9) | 323 | 0.945 |  |
| Densitybased(K=9) | 318 | 0.946 |  |

**Structural variation across body habitats**: Our goal is to utilize the results of different clustering results and generate a more reliable result. Through analyzing the enrichment of body habitat and host gender over six predicted clusters generated by four base clustering algorithms, the results in Figure S3 revealed a stronger coherence by body habitat than host gender. Similar to modularity structure of Meta-EC, K-Mean and Density-based clustering generated one oral cavity cluster, two gut clusters differentiated by gender, three skin clusters dominated by male individuals, while Hierarchical and EM clustering produced one gut cluster, one oral cavity cluster and four skin-dominated clusters. In particular, three of skin clusters detected by EM algorithm is quite similar with skin clusters by K-Mean and Density-based clustering in terms of habitat and gender sample distribution in clusters, while remaining one skin clusters are impure with 32% gut samples and 23% oral cavity samples.

Finally, our ensemble framework combines the co-clustering relationship that is identified by these clustering results and learns the modular structure from consensus matrix. Basically, co-clustering relationship on consensus matrix are assigned higher confidence scores if the co-clustering relationships are identified by more clustering approaches. The results in Figure 9 shows that Meta-EC is able to capture the reliable clusters according to consensus result of base clustering results, therefore filtering out unreliable ones with high impurity.

For structural variation over host gender in oral cavity, gut and skin-dominated clusters, the consensus results of base clustering algorithm could be captured by that of Meta-EC, therefore we only present the host gender variation of ensemble results.

**
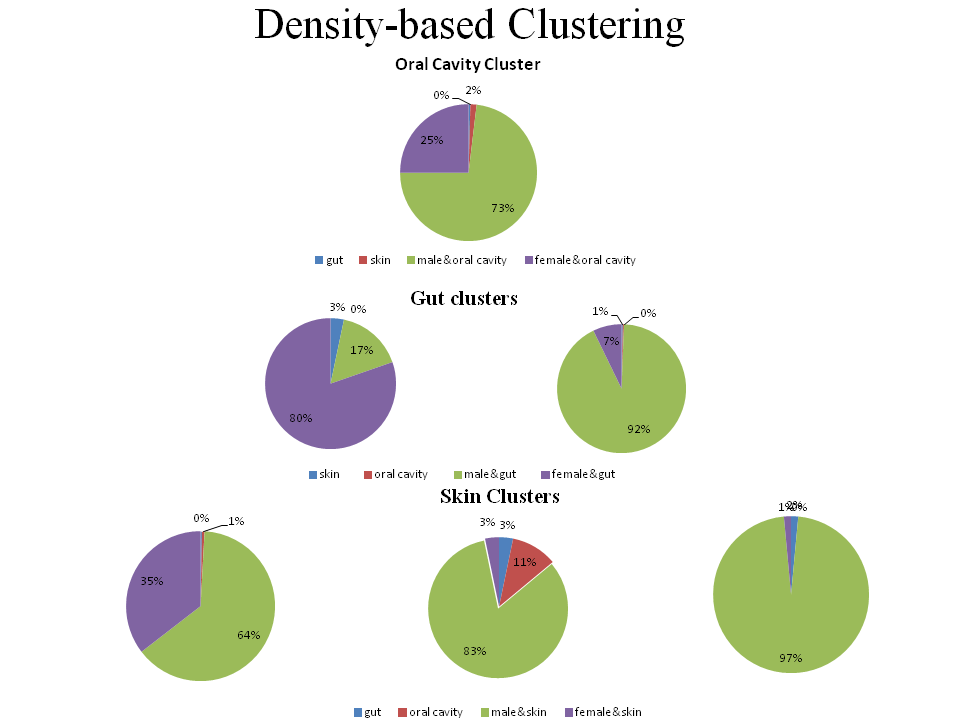
**

**
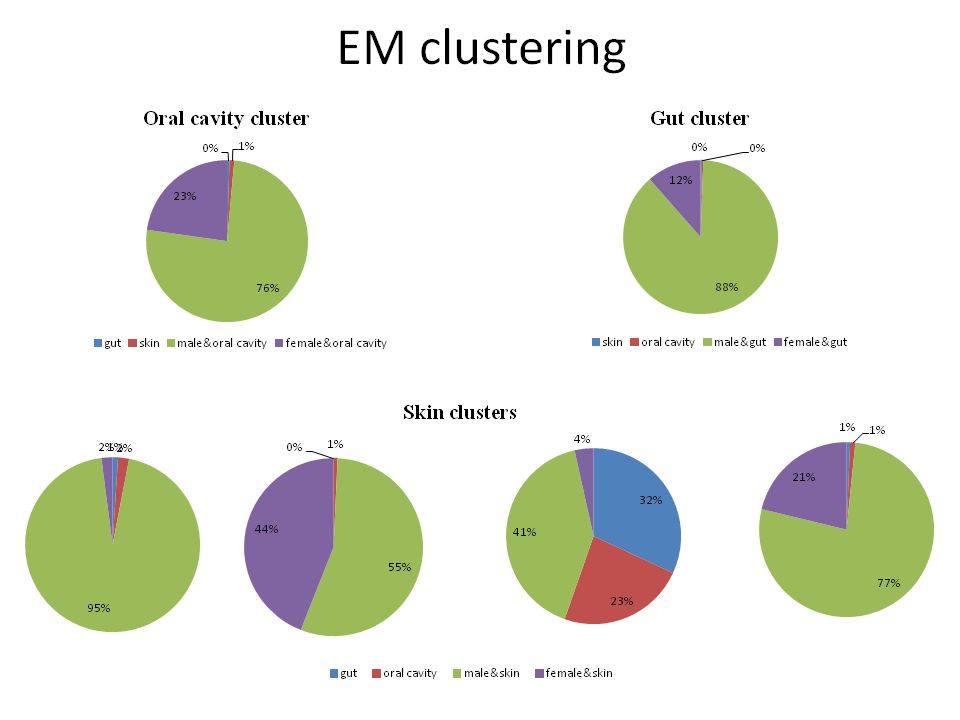
**

**
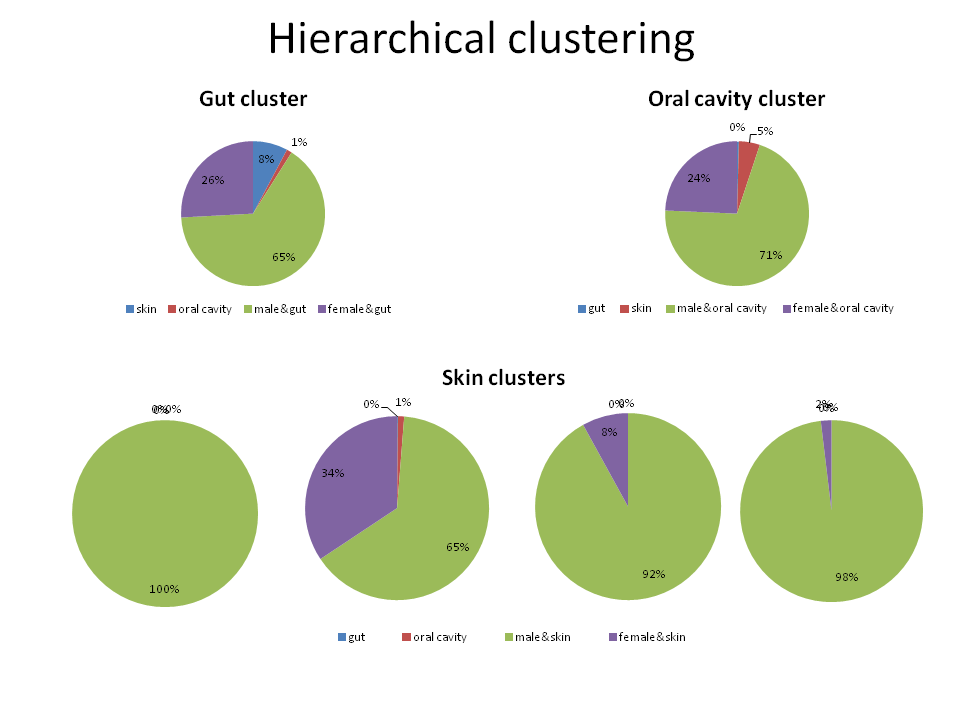
**

**
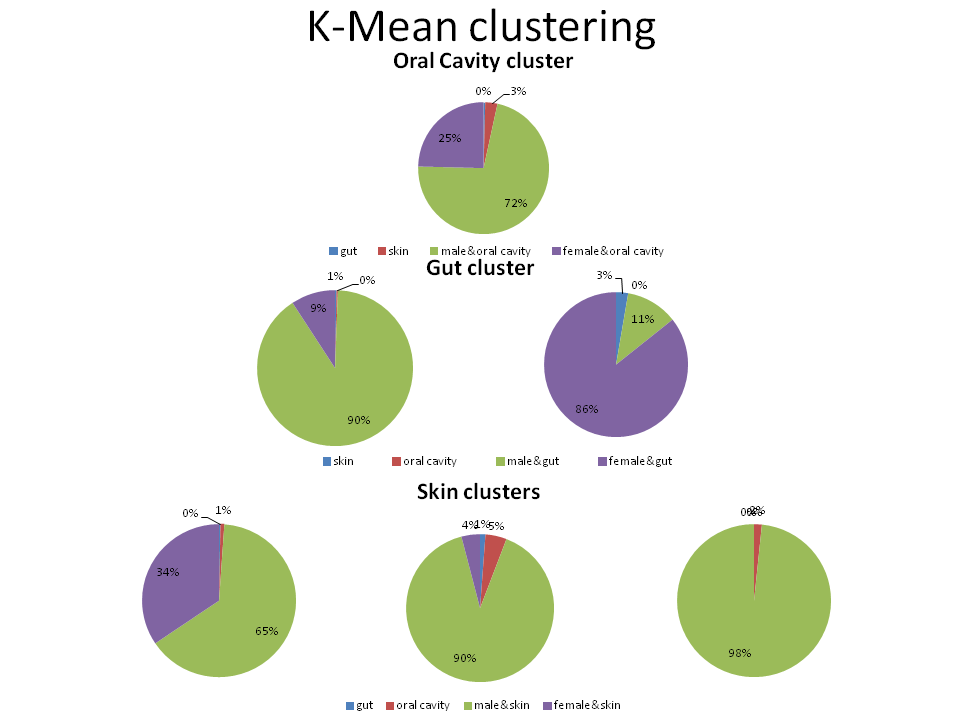
**

**Figure S3: Sample distribution on predicted clusters with respect to body habitat and host gender**

1. **Sensitivity study of phylogenetic structure similarity on microbial network**

Recall that phylogenetic structure similarity is a “soft” structure similarity relationship between two metagenomic samples in network *G*. Higher similarity value indicates similar phylogenetic structure between microbial communities. Since the clustering results are generated on the metagenomic similarity network, the quality of the metagenomic similarity affects the performance of microbial community identification. Therefore, we conduct sensitivity study of phylogenetic similarity of edge set *E* in metagenomic network *M*. In sensitivity study, a threshold for edge set *E* is set to retain the edges with similarity values higher than predefined threshold value. Basically, a lower threshold value of *E* will include many noisy and un-relevant relationships in network *G* while a higher threshold value of *E* will reduce number of useful relationships and may miss out a few important relevant relationships as a result.

To study the effect of the threshold of *E* on microbial community identification, we ran algorithm Meta-EC with threshold value tuning from 0.7 to 0.9 with 0.1 as step size. Clustering performances are evaluated by the three measurements corresponding to reference communities. Results are shown in **Figure S4**. For ensemble clustering with random initialization, the value of $\beta$ is set to 0.5, 8, 32 for threshold value 0.7, 0.8 and 0.9 respectively. The performance of our algorithm improved with increasing threshold value of 0.7, indicating that excluding low phylogenetic similarity will help reduce noisy and un-meaningful associations of metagenomic samples in network *G*, which is helpful to improve microbial community detection. However, if we further exclude more edges with high phylogenetic similarity, meaningful and important sample association will be removed and eventually affects the performance of microbial community identification. As shown in **Figure S4**, the performance within range from 0.8 to 0.9 has worsened. In addition, none of these similarity networks achieves superior results in terms of all three measurements, showing that microbial community patterns with inconsistent phylogenetic structures are involved in environmental conditions. Nevertheless, the ensemble algorithm performance of community detection consistently outperformed other state-of-art clustering techniques in the wide range of edge threshold, indicating that our algorithm is robust and insensitive to the similarity network noisy and data coverage.


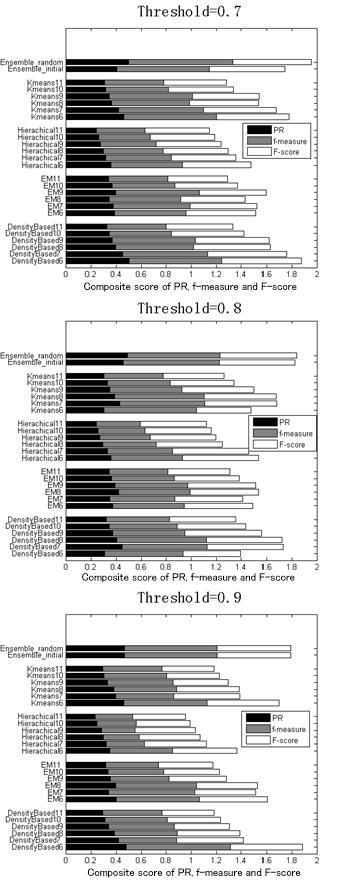


**Figure S4: Sensitivity study of phylogenetic similarity on the metagenomic network.** Additive values of three measures (PR, *f*-measure and F-score) are present for ensemble framework and four base clustering approaches.

**Reference**

1. Su X, Xu J, Ning K: Meta-Storms: efficient search for similar microbial communities based on a novel indexing scheme and similarity score for metagenomic data. *Bioinformatics* 2012, **28**(19): 2493-2501.
