# Supplementary material for: Microbial community pattern detection in human body habitats via ensemble clustering framework
Source: BMC Syst Biol. 2014 Dec 8;8(Suppl 4):S7. doi: 10.1186/1752-0509-8-S4-S7 (PMC4290728; doi:10.1186/1752-0509-8-S4-S7)
Supplement: Additional file 1 — Experimental Design. The file show the experimental design in this paper, including: (1) introductory of four base clustering approaches; (2) evaluation of microbial clusters; (3) parameter setting. [file 1752-0509-8-S4-S7-S1.docx]

**Experimental Design**

## Microbial community pattern detection in human body habitats via ensemble framework

Peng Yang, Xiaoquan Su, Le Ouyang, Hon-Nian Chua, Xiao-Li Li, Kang Ning^§^

^§^Corresponding author

Kang Ning: email: [ningkang@qibebt.ac.cn](mailto:ningkang@qibebt.ac.cn)

1. **Introductory of four base clustering approaches**

The goal of ensemble clustering is to obtain a comprehensive consensus clustering by integrating *m* component clustering results. Given the metagenomic similarity network, there are several ways to obtain diverse clustering results. They can be generated by different clustering algorithms as well as a given algorithm with different initializations. These clustering results should be generated by diverse and independent computational approaches, called base clustering algorithms.

In our experiment, proposed ensemble algorithm Meta-EC is composed of four base clustering approaches:

**HIERARCHICAL CLUSTERING** builds clusters based on distance connectivity [[1](#_ENREF_19)]. Initially, each vertex in *V* is represented as a cluster, namely $\left\{ v_{1} \right\}\ldots\left\{ v_{n} \right\}$, where $v_{i}\epsilon V$ and *n* is number of vertex in *V*. In each iteration of hierarchical clustering, the algorithm chooses two closest clusters to merge in a new cluster. Given two clusters A and B, the distance between two clusters can be defined in many ways. Here we use the popular average linkage, which captures the average distance between two sets of vertices: $\frac{1}{|A|\cdot|B|}\sum_{v_{i}\epsilon A} \sum_{v_{j}\epsilon B} d(v_{i},v_{j})$. The algorithm stops clustering when the number of merged clusters reaches a sufficiently small number.

**K-MEANS CLUSTERING** aims to partition the vertexes into *k* clusters where each vertex in identical clusters has the nearest mean [[2](#_ENREF_20)]. In *k*-means algorithm, the similarity network *G* is represented as its matrix format, i.e. $W = [w_{ij}]$ where $w_{ij}$ equals to the similarity between vertex $v_{i}$ and $v_{j}$. In this way, each vertex $v_{i}$ in *V* is represented as a *n*-dimensional real vector, namely $\vec{v}_{i}=\{w_{i1},\ldots,w_{in}\}$. Given *k* cluster set $=\{S_{1},\ldots,S_{k}\}$ with randomly initialized points, *k*-means clustering aims to partition each vertex $\vec{v}_{i}$ into k clusters in order to minimize the similarity as: $\arg\min_{S} \sum_{i=1}^{k} \sum_{\vec{v}_{j}\epsilon S_{i}} \left\| \vec{v}_{j}-\vec{\mu}_{i} \right\|^{2}$, where $\vec{\mu}_{i}$ is the mean of points in $S_{i}$.

**EM ALGORITHM** assigns a probability distribution to each vertex to indicate the likelihood it belongs to each of the clusters [[2](#_ENREF_20)]. Given the similarity network consisting of a vertex set *V* and *k* initialized cluster set $\left\{ \mu_{1} \right\}\ldots,\{\mu_{k}\}$, where $\{\mu_{j}\}$ is a random initial cluster point, EM algorithm repeats the following two steps to re-estimate cluster set until each cluster point $\mu_{j}$ converges to a steady state:

Expectation step *(E step*): compute expectation value of each vertex $\vec{v}_{i}$ that belongs to cluster point $\{\mu_{j}\}$, denoted as $E\left[ Z_{ij} \right]=\frac{p(v=\vec{v}_{i}|\mu=\mu_{j})}{\sum_{h=1}^{k} p(v=\vec{v}_{i}|\mu=\mu_{h})}$;

Maximization step (*M step*): re-compute $\left\{ \mu_{1^{'}} \right\}\ldots\left\{ \mu_{k^{'}} \right\}$ as $\left\{ \mu_{j^{'}} \right\}\leftarrow\frac{\sum_{i=1}^{n} E\left[ Z_{ij} \right]\vec{v}_{i}}{\sum_{i=1}^{n} E\left[ Z_{ij} \right]}$, where *n* is number of vertexes in *V*.

**DENSITY-BASED CLUSTERING** defines clusters as areas of high local density relative to the rest of the network space [[3](#_ENREF_21)]. A typical example in this category is CFinder which detects the k-clique percolation clusters as functional modules. A parameter $\varepsilon$ is required in the algorithm. The algorithm randomly prioritizes an unvisited vertex $v_{i}$. If the vertex $v_{i}$ has sufficient sized $\varepsilon$-neighborhood, a cluster is generated. If one vertex $v_{j}$ is found to be a dense part of $v_{i}$’s cluster, all vertexes in $v_{j}$’s $\varepsilon$-neighborhood add to $v_{i}$’s cluster. This process continues until the density-connected cluster is completely found. Then a new unvisited vertex is retrieved and processed.

1. **Evaluation of microbial clusters**

To evaluate the accuracy of different clustering methods in grouping sample with same features, we introduce three scoring measures to assess how well the detected clusters match the reference communities. We first introduce some notations before describing these measures. Let $C=\{C_{1}, C_{2}, \cdots,C_{K}\}$ denotes the clusters detected by a particular clustering algorithm, where each $C_{z}$, $z=1,\cdots,K$ is consist of samples in the z-th cluster and $\left| C \right|=K$ denotes the number of clusters. Let $F=\{F_{1}, F_{2}, \cdots,F_{T}\}$ denotes the reference set, where each $F_{t}, t=1, \cdots,T$ is consist of samples within the t-th reference community. $\left| F \right|=T$ denotes the number of reference communities.

1. **PR-based metric**: For each cluster z and each reference community t, the Precision-based score between them is calculated by ${Pre}_{zt}= \frac{|C_{z} \cap F_{t}|}{|C_{z}|}$, which measures what fraction of the samples in cluster z correspond to reference community t. The Recall-based score between them is calculated by ${Rec}_{zt}= \frac{|C_{z} \cap F_{t}|}{|F_{t}|}$ which measures what fraction of the reference community t is recovered by cluster z. The Precision-Recall based score is defined as ${PR}_{zt}={Pre}_{zt}\times{Rec}_{zt}$. For each cluster z, we try to find the reference community that maximizes the Precision-Recall based score between them, which is defined as ${PRC}_{z}={max}_{t} {PR}_{zt}$. Similarly, for each reference community t, we try to find the cluster that maximizes the Precision-Recall based score between them, that is ${PRF}_{t}={max}_{z} {PR}_{zt}$. After measuring ${PRC}_{z}$ for the each detected clusters, we define $\boldsymbol{PRC}= \frac{\sum_{z=1}^{K} {PRC}_{z}}{K}$. Similarly, for the T reference communities, we define $\boldsymbol{PRF}= \frac{\sum_{t=1}^{T} {PRF}_{t}}{T}$. To quantify how the K detected clusters overlap with the T reference communities, the PR between detected clusters and reference communities is defined as the harmonic mean of ***PRC*** and ***PRF***, which is

$PR= \frac{2\times PRC\times PRF}{PRC+PRF}$ (1)

1. ***f*-measure**: Another evaluation metric is *f*-measure which is also based on precision and recall, but the definition of precision and recall is different from PR-based metric. *f*-measure assesses the performance of a clustering algorithm at cluster level, whereas PR-based metric is at cluster-sample level. For a detected cluster z and a reference community t, they are considered to be matched if ${PR}_{zt}\geq\rho$, here $\rho$ is a predefined threshold, which is generally set to 0.25 in the literature. So in this paper, the value of $\rho$ is fixed at 0.25. Let N be the number of detected clusters that match at least one reference community and M be the number of reference communities that are matched by at least one detected clusters. The precision is defined as $Precision= \frac{N}{K}$, and the recall is defined as $Recall= \frac{M}{T}$. The *f*-measure is defined as the harmonic mean of recall and precision, that is

$f-measure= \frac{2\times Precision\times Recall}{Precision+Recall}$ (2)

1. **F-score**: The third evaluation metric is F-score, which measures the performance of a clustering algorithm at sample level. Let TT denotes the number of sample pairs that belong to same reference communities. Let PP denotes the number of sample pairs that are grouped together by a given clustering algorithm. Let TP denotes the number of sample pairs occur simultaneously in same reference communities and same detected clusters. In this way, precision-based score could be defined as $pre=\frac{TP}{PP}$, whereas recall-based score is defined as $rec=\frac{TP}{TT}$. Finally, F-score is defined as

$F-score=\frac{2\times pre\times rec}{pre+rec}$ (3)

1. **Parameter setting**

Ensemble learning algorithm strives to combine many components of the network *G* into a more reliable clustering result. These components can be obtained from a base clustering algorithm with different initializations or from different clustering algorithms on a network.

Clustering results are generated by base algorithms. Assuming that there are about one or two microbial structural patters within six particular habitats, we set number of generated clusters from 6 to 11. Our expectation is to find out all possible structural patterns within one habitat, therefore increasing cluster number for base algorithm may find out small sized clusters within one microbial habitat. Sensitive study has been performed to number of generated clusters in four base algorithms in **Figure 5**. The results show that it achieve better performance when generated cluster is set to 6 while higher number from 9 to 11 may lead to many small-sized cluster patterns that were far from referred meta-data of our samples and lead to poor performance on three measures. Hence, our predefined cluster number region from 6 to 11 is sufficient to identify the microbial structure patters in this study.

Four state-of-art algorithms, Base clustering algorithms, including EM algorithm, K-mean clustering, hierarchical clustering and density-based clustering, have been used as base algorithms and applied on metagenomic matrix to generate base clustering results. Hierarchical clustering computes the cluster distance with ‘COMPLETE’ linktype, which considers the distance of all possible sample pairs between two clusters. K-mean clustering, density-based clustering and hierarchical clustering measure sample pair similarity with Euclidean distance. All base algorithms set maximal iteration with 500, minimal standard deviation with 1.0E-6 and starting seeds with 100 in the experiments.

When we combine base clustering results into one ensemble matrix, we apply the symmetric NMF-based clustering algorithm on the matrix to derive the clusters. In this step, there are three parameters need to predefined, the first one is the number of possible clusters *K*, the second one is the hyper-parameter $\beta$ and the last one is the threshold $\tau$. $\tau$ is the threshold parameter to obtain the sample-cluster membership matrix. We find experimentally that $\tau=0.3$ always corresponds to reasonable performance, thus, we fix the value of $\tau=0.3$. Since the true number of communities is previously unknown, how to decide the number of clusters is still an open problem. However, on one hand, given several base clustering results, we could choose the one with the best performance as the initial input for the ensemble-based approach. In this way, the number of possible clusters *K* is the number of clusters of the initial input. On the other hand, if there is not any prior information, we could use random initialization as the input of our algorithm. In this case, the value of *K* could be set to relatively large since in our algorithm, the final result is obtained by filtering out irrelevance clusters through threshold $\tau$. Furthermore, we experimentally find that given a base clustering result as an initial input, the performance of our algorithm is not very sensitive to the choice of $\beta$. Thus, we fix the value of $\beta$ to be 1 in these cases. With random initialization, the value of $\beta$ may affect the performance of our algorithm. Therefore, we choose the value of $\beta$ from 2^-5^ to 2^5^, and selected the one which corresponds to the best performance.

**Reference**

1. Szekely GJ, Rizzo ML: **Hierarchical clustering via Joint Between-Within Distances: Extending Ward's Minimum Variance Method**. *Journal of classification* 2005, **22**(2): 151-183.

2. Lloyd SP: **Least squares quantization in PCM**. IEEE Transactions on Information Theory 1982, **28**:129-137.

3. Dempster AP, Laird NM, Rubin DB: **Maximum Likelihood from Incomplete Data via the EM Algorithm**. *Journal of the Royal Statistical Society* 1977, **39**:1–38.
